# Molecular Characterization of the *viaB* Locus Encoding the Biosynthetic Machinery for Vi Capsule Formation in *Salmonella* Typhi

**DOI:** 10.1371/journal.pone.0045609

**Published:** 2012-09-21

**Authors:** Michael Wetter, David Goulding, Derek Pickard, Michael Kowarik, Charles J. Waechter, Gordon Dougan, Michael Wacker

**Affiliations:** 1 GlycoVaxyn AG, Schlieren, Switzerland; 2 Wellcome Trust Sanger Institute, Wellcome Trust Genome Campus, Cambridge, United Kingdom; 3 Department of Molecular and Cellular Biochemistry, University of Kentucky College of Medicine, Lexington, Kentucky, United States of America; University of Helsinki, Finland

## Abstract

The Vi capsular polysaccharide (CPS) of *Salmonella enterica* serovar Typhi, the cause of human typhoid, is important for infectivity and virulence. The Vi biosynthetic machinery is encoded within the *viaB* locus composed of 10 genes involved in regulation of expression (*tviA*), polymer synthesis (*tviB-tviE*), and cell surface localization of the CPS (*vexA-vexE*). We cloned the *viaB* locus from *S*. Typhi and transposon insertion mutants of individual *viaB* genes were characterized in *Escherichia coli* DH5α. Phenotype analysis of *viaB* mutants revealed that *tviB*, *tviC*, *tviD* and *tviE* are involved in Vi polymer synthesis. Furthermore, expression of *tviB-tviE* in *E. coli* DH5α directed the synthesis of cytoplasmic Vi antigen. Mutants of the ABC transporter genes *vexBC* and the polysaccharide copolymerase gene *vexD* accumulated the Vi polymer within the cytoplasm and productivity in these mutants was greatly reduced. In contrast, *de novo* synthesis of Vi polymer in the export deficient *vexA* mutant was comparable to wild-type cells, with drastic effects on cell stability. *VexE* mutant cells exported the Vi, but the CPS was not retained at the cell surface. The secreted polymer of a *vexE* mutant had different physical characteristics compared to the wild-type Vi.

## Introduction

The causative agent of the human systemic infection typhoid fever, *Salmonella enterica* subspecies I serotype Typhi (*S*. Typhi), expresses a capsular polysaccharide (CPS) known as Vi antigen (Vi). The Vi capsule provides *S*. Typhi with mechanisms to avoid host defenses [1], [2] and it is important in enhancing infectivity and virulence [3], [4], [5]. Besides being an important virulence factor, the Vi is also a protective antigen and a vaccine based on purified Vi polysaccharide has been developed and licensed for use as a parenteral vaccine against typhoid fever [6].

**Figure 1:**
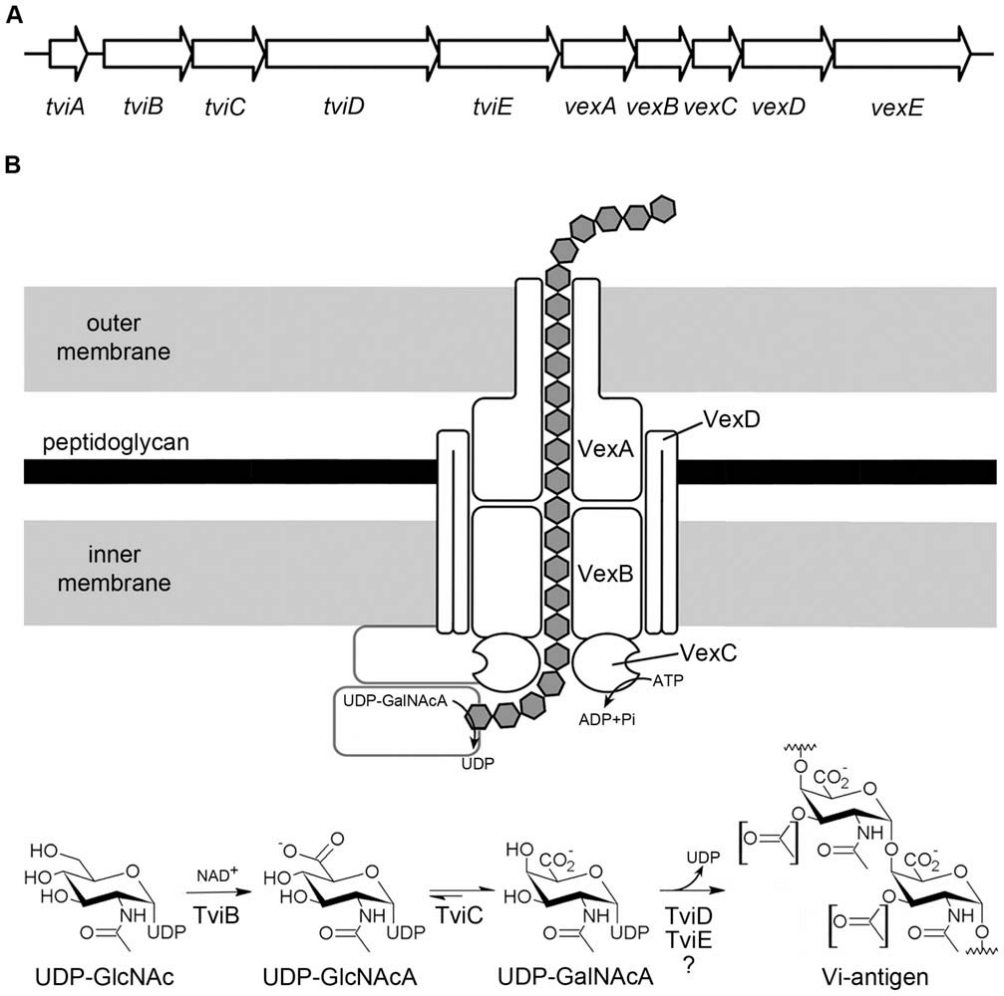
Overview of the *viaB* operon and Vi capsular polysaccharide biosynthesis pathway. (**A**) Schematic diagram of the *S*. Typhi *viaB* operon. (**B**) The Vi antigen is a linear polymer of α-1,4-linked *N*-acetylgalatosaminuronate, nonstoichiometrically esterified with acetyl groups at the C-3. A TviB-catalyzed oxidation of UDP-*N*-acetylglucosamine (UDP-GlcNAc) followed by the TviC-catalyzed epimerization at the C-4 of UDP-*N*-acetylglucosaminuronate (UDP-GlcNAcA) results in the formation of UDP-*N*-acetylgalactosaminuronate (UDP-GalNAcA), the building block for Vi polymer formation. The Vi polymer is synthesized in the cytoplasm and assembly is dependent on TviD and TviE. The enzyme catalyzing the O-acetylation of the capsular polysaccharide has not yet been identified. Subsequent translocation of the polysaccharide to the cell surface follows an ATP-binding cassette (ABC) transporter-dependent process. The transporter consists of VexA, VexB, VexC and VexD. The precise function of VexE is equivocal although it might be involved in anchoring the Vi to the cell surface.

Vi is a linear, acidic homopolymer of α-1,4-linked *N*-acetylgalactosaminuronate (D-GalNAcA), variably O-acetylated at C-3. The O-acetyl groups make up most of the surface and the immunogenicity of Vi is closely related to the degree of O acetylation [7], [8]. Key proteins involved in Vi capsule formation are encoded within a cluster of genes, known historically as the *viaB* locus [9], a region located on a 134 kb DNA island, termed *Salmonella* pathogenicity island 7 (SPI-7) within *S*. Typhi [10]. The *S*. Typhi *viaB* constitutes 10 genes involved in regulation of expression (*tviA*), biosynthesis (*tviB* to *tviE*), and cell surface localization of the Vi polysaccharide (*vexA* to *vexE*) (Figure 1A). Analysis of the bioinformatics signature of *viaB* highlighted the presence of a putative ATP-binding cassette (ABC) transporter and the absence of a homologue of *wzy*/*wzx* respectively. Therefore, biosynthesis of Vi is thought to be similar to *E. coli* group 2 CPS [11].

Vi expression in *S*. Typhi is under control of the RcsB-RcsC and OmpR-EnvZ two-component regulator systems [12], [13], [14] and when the complete *viaB* operon is expressed in *Escherichia coli* K12 a similar pattern of regulation can also be observed [15]. RcsB-RcsC and OmpR-EnvZ likely interact with TviA and regions upstream of the *tviA* promoter thereby linking Vi expression to environmental signatures such as osmolarity [16]. TviA is an activator of the *viaB* operon and deletion of the *tviA* gene strongly decreases expression of the Vi capsule [17].

Biosynthesis of the Vi polysaccharide takes place in the cytoplasm and requires functional TviB, TviC, TviD and TviE proteins [17] (Figure 1B). TviB and TviC are involved in catalyzing the conversion of UDP-*N*-acetylglucosamine (UDP-GlcNAc) to UDP-*N*-acetylgalactosaminuronate (UDP-GalNAcA), which serves as the building block for the assembly of the Vi polymer [18]. Assembly of the Vi polymer is dependent on functional expression of *tviD* and *tviE* [17]. The enzyme responsible for O-acetylation of the Vi polysaccharide has not yet been identified.

Cell surface localization of the Vi polymer is dependent on functional expression of *vexA*, *vexB*, *vexC*, *vexD* and *vexE* (Figure 1B). The predicted lipoprotein VexA belongs to group B of the outer membrane polysaccharide export (OPX) proteins [19]. OPX proteins for which a high-resolution structures have been solved include Wza [20], a protein essential for group 1 CPS expression on the surface of *E. coli* and the *E. coli* group 4 capsule protein GfcC [21]. The Wza protein forms an octameric structure that spans the outer membrane and protrudes into the periplasm, thereby forming a water-filled channel. One of the most extensively studied bacterial group 2 capsules is the K1 serotype of *E. coli*. The *E. coli* K1 capsular gene cluster encodes KpsD, which is the functional homologue of VexA. VexBC belong to the large family of ABC transporters whose members have been implicated in the transport of substrates across membranes. The *E. coli* K1 ABC transporter KpsMT is a close homologue of VexBC [22]. KpsM is a hydrophobic, integral inner membrane protein with six transmembrane domains whereas KpsT is a hydrophilic, peripheral inner membrane protein containing an ATP-binding domain. The functional transporter is proposed to consist of two subunits each of KpsM and KpsT. VexD and its homologous protein KpsE of *E. coli* K1 belong to a family called the polysaccharide copolymerases subfamily 3 (PCP-3) [19]. There is no structural information yet available about this subfamily but all PCP proteins have a characteristic membrane topology in which a large periplasmic loop is flanked by two transmembrane regions localized in the inner membrane. PCP-3 proteins might provide a periplasmic scaffold for linking the ABC transporter in the inner membrane with the OPX protein in the outer membrane therefore assembling the complete polysaccharide translocation machinery. VexE seems to be responsible for anchoring the Vi to the cell surface [17].

To investigate the function of individual proteins involved in biosynthesis and cell surface expression of the Vi CPS in greater detail, the *viaB* cluster was cloned from *S*. Typhi and transposon insertion mutants of individual *viaB* genes were characterized in *E. coli* DH5α. A detailed and comprehensive phenotype characterization of *viaB* single gene mutants and their effect on biosynthesis and cell surface expression of the Vi CPS is reported here.

## Results

### Expression of the Vi capsule in E. coli DH5α

The low copy plasmid pGVXN158 was constructed using a 14.9 kb DNA fragment of *S*. Typhi BRD948 that harbors the 10 open reading frames of the *viaB* operon with around 900 bp upstream of the first gene *tviA*. Therefore the *viaB* operon contains the natural regulatory sequences and expression is not controlled by elements encoded on the plasmid backbone. *E. coli* DH5α was transformed with pGVXN158 whereupon the transformants changed colony morphology towards a smooth colony appearance, indicating the expression of a capsule. These cells could be agglutinated using a Vi specific antibody. Furthermore, these encapsulated cells were tested for susceptibility to infection by well characterized Vi phages that are part of the classical typing set [23], [24], [25], [26], [27]. All 7 Vi phage types were able to infect *E. coli* DH5α(pGVXN158) but not the plasmid free controls. Thus, using these simple methodologies the Vi produced by *E. coli* was indistinguishable from Vi expressed by the parenteral *S*. Typhi. Therefore, this Vi expression system harbored in *E. coli* DH5α was used for further analysis of the biosynthetic machinery assembling the Vi capsule.

### Quantification and localization of the Vi in E. coli DH5α(pGVXN158) harboring single gene transposon insertions within viaB genes

**Figure 2:**
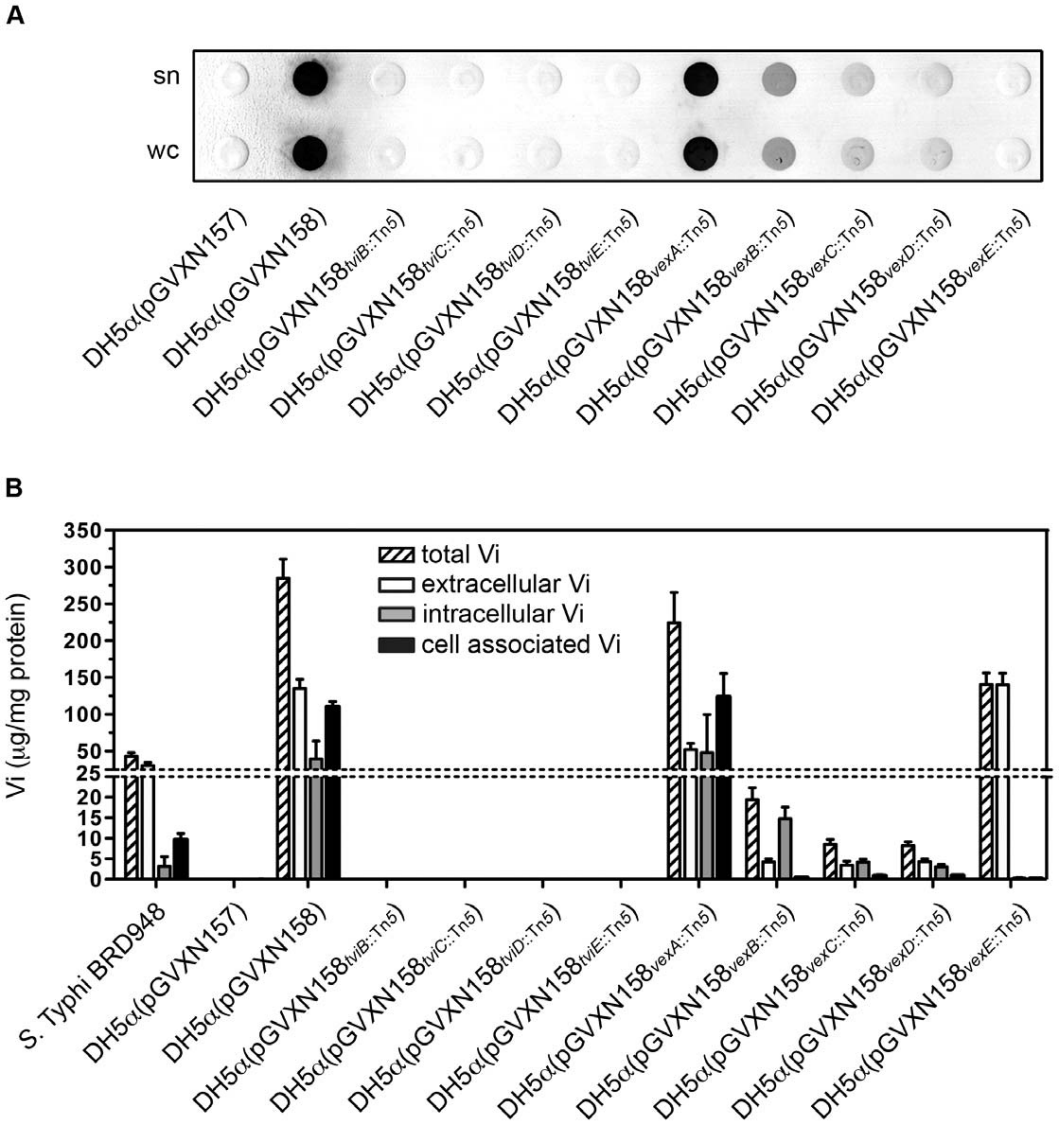
Localization and Quantification of the Vi polysaccharide in different viaB transposon insertion mutants. (**A**) Washed cell pellets (wc) and the corresponding supernatants (sn) of DH5α overnight cultures harboring the indicated plasmids were spotted onto nitrocellulose and blotted with anti-Vi antiserum. (**B**) Vi content was measured in cell culture supernatants (extracellular Vi), in a suspension of washed and fixed cells (cell associated Vi), and in cell lysates using a quantitative sandwich ELISA. Intracellular Vi was defined using the difference in Vi measured in cell lysates and fixed cells. The amount of Vi was normalized to the protein content measured in the cell pellets.

Next the role of the individual genes of the *viaB* operon in Vi capsule expression was analyzed. For this purpose single genes within the *viaB* operon encoded on pGVXN158 were inactivated by Tn*5*-mediated transposon insertional mutagenesis. To this end, *viaB* single gene insertion mutants in all of the genes except the regulator *tviA* were selected and the insertion site was mapped (Table 1). Washed cell pellets and the corresponding supernatants of overnight cultures from individual transposon insertion mutants were spotted onto nitrocellulose membranes and the presence of the Vi CPS was detected by immunoblotting using a monoclonal Vi specific antibody. To exclude polar effects on downstream genes, *tviB*::Tn5, *tviC*::Tn5, *tviD*::Tn5, *tviE*::Tn5, *vexB*::Tn5, *vexC*::Tn5 transposon insertion mutants were complemented with the corresponding gene cloned and Vi expression was restored (data not shown). Detection of Vi polysaccharide in the individual *E. coli* DH5α(pGVXN158) transposon mutant derivatives revealed three distinguishable phenotypes (Figure 2A): i) a strong signal was detected with cell cultures from *E. coli* DH5α(pGVXN158) and *E. coli* DH5α(pGVXN158) harboring *vexA*::Tn*5* (pGVXN158*_vexA_*_::Tn*5*_) ii) an intermediate staining was associated with *vexB*::Tn*5* (pGVXN158*_vexB_*_::Tn*5*_), *vexC*::Tn*5* (pGVXN158*_vexC_*_::Tn*5*_), and *vexD*::Tn*5* (pGVXN158*_vexD_*_::Tn*5*_), whereas iii) no Vi was detected in cell cultures of *E. coli* DH5α carrying the empty plasmid control pGVXN157 or *E. coli* DH5α(pGVXN158) harboring *tviB*::Tn*5* (pGVXN158*_tviB_*_::Tn*5*_), *tviC*::Tn*5* (pGVXN158*_tviC_*_::Tn*5*_), *tviD*::Tn*5* (pGVXN158*_tviD_*_::Tn*5*_), *tviE*::Tn*5* (pGVXN158*_tviE_*_::Tn*5*_) or *vexE*::Tn*5* (pGVXN158*_vexE_*_::Tn*5*_). To determine if the detection of Vi observed in the *vexA*::Tn*5*, *vexB*::Tn*5*, *vexC*::Tn*5* or *vexD*::Tn*5* transposon insertion mutants was associated with surface expression of capsular Vi, slide agglutination and Vi phage infections were performed (Table 1). None of the cells harboring these transposon insertion mutants could be agglutinated with a Vi specific antibody and none of the tested mutants could be infected with either one of the seven Vi phages. Taken together these results indicate that Vi polysaccharide is still produced in cells expressing the *viaB* locus with a transposon insertion in *vexA*, *vexB*, *vexC* and *vexD* but this is not cell surface associated.

**Table 1:** Overview of transposon insertion mutants analyzed in this study.

Plasmid	Tn*5* insertion site*	Immunoblotting	Agglutination	Phage infection**
pGVXN157	−	−	−	−
pGVXN158	−	+++	+	+
pGVXN158*_tviB_*_::Tn*5*_	679/1278	−	−	nd
pGVXN158*_tviC_*_::Tn*5*_	268/1047	−	−	nd
pGVXN158*_tviD_*_::Tn*5*_	1909/2496	−	−	nd
pGVXN158*_tviE_*_::Tn*5*_	388/1737	−	−	nd
pGVXN158*_vexA_*_::Tn*5*_	350/1068	++	−	−
pGVXN158*_vexB_*_::Tn*5*_	679/795	+	−	nd
pGVXN158*_vexC_*_::Tn*5*_	515/696	+	−	−
pGVXN158*_vexD_*_::Tn*5*_	820/1305	+	−	nd
pGVXN158*_vexE_*_::Tn*5*_	1247/1971	−	−	−

* bp of insertion site from 3′ end of the gene/total length of gene.

** nd: not determined.

To generate a more detailed view about the quantities and the localization of the Vi polysaccharide produced in the different *viaB* insertion mutants, a sandwich ELISA was developed. With this method Vi polysaccharide contents were measured in the cell culture supernatant (extracellular Vi), in a suspension of washed cells that were fixed with paraformaldehyde (cell associated Vi), and in cell lysates. Intracellular Vi was defined using the difference in Vi measured in cell lysates and fixed cells (Figure 2B).

*E. coli* DH5α(pGVXN158) cells expressed 6–7 times more Vi than *S*. Typhi BRD948, although the cellular distribution of Vi was similar. Vi was mainly detected on the cell surface, and in the cell culture supernatant. No Vi was detected in DH5α(pGVXN158*_tviB_*_::Tn*5*_), DH5α(pGVXN158*_tviC_*_::Tn*5*_), DH5α(pGVXN158*_tviD_*_::Tn*5*_), DH5α(pGVXN158*_tviE_*_::Tn*5*_) or DH5α(pGVXN157). These data confirm the results by Virlogeux et al. [17] that TviB, TviC, TviD and TviE are involved in the biosynthesis of the Vi polysaccharide. Vi distribution looked similar in DH5α(pGVXN158*_vexB_*_::Tn*5*_), DH5α(pGVXN158*_vexC_*_::Tn*5*_) or DH5α(pGVXN158*_vexD_*_::Tn*5*_) whereas these insertion mutants produced 15–35 times less Vi in total as compared to DH5α(pGVXN158). Vi in these mutants was mainly detected predominantly inside the cells and in the culture supernatants. In contrast, DH5α(pGVXN158*_vexA_*_::Tn*5*_) and DH5α(pGVXN158*_vexE_*_::Tn*5*_) produced large amounts of Vi the former in the same magnitude range as DH5α(pGVXN158) whereas the *vexE*::Tn*5* mutant produced approximately half the amount of the total Vi measured in DH5α cells expressing the wild type *viaB* locus. However, cellular distribution of the Vi in these two insertion mutants was different. In DH5α(pGVXN158*_vexA_*_::Tn*5*_) cultures, slightly lower amounts of Vi were detected in the cell culture supernatant, whereas comparable amounts of intracellular and cell associated Vi was measured. The detection of surface exposed Vi in this *viaB* insertion mutant is potentially in conflict with the observed resistance of this strain to Vi phage infection and the fact that it is not agglutinable with a Vi antigen specific antibody. The Vi produced by cells harboring a *vexE* transposon insertion plasmid was mainly localized in the cell culture supernatant with similar amounts comparable to DH5α(pGVXN158). However, in contrast to DH5α(pGVXN158), only low levels of intracellular and cell surface associated Vi was detectable. This result indicates that the *vexE* mutant expresses a functional Vi translocation machinery, but once the polymer is exported, the Vi is not efficiently retained on the cell surface.

### Characterization of vex transposon insertion mutants by transmission electron microscopy and immunogold labeling of the Vi

**Figure 3:**
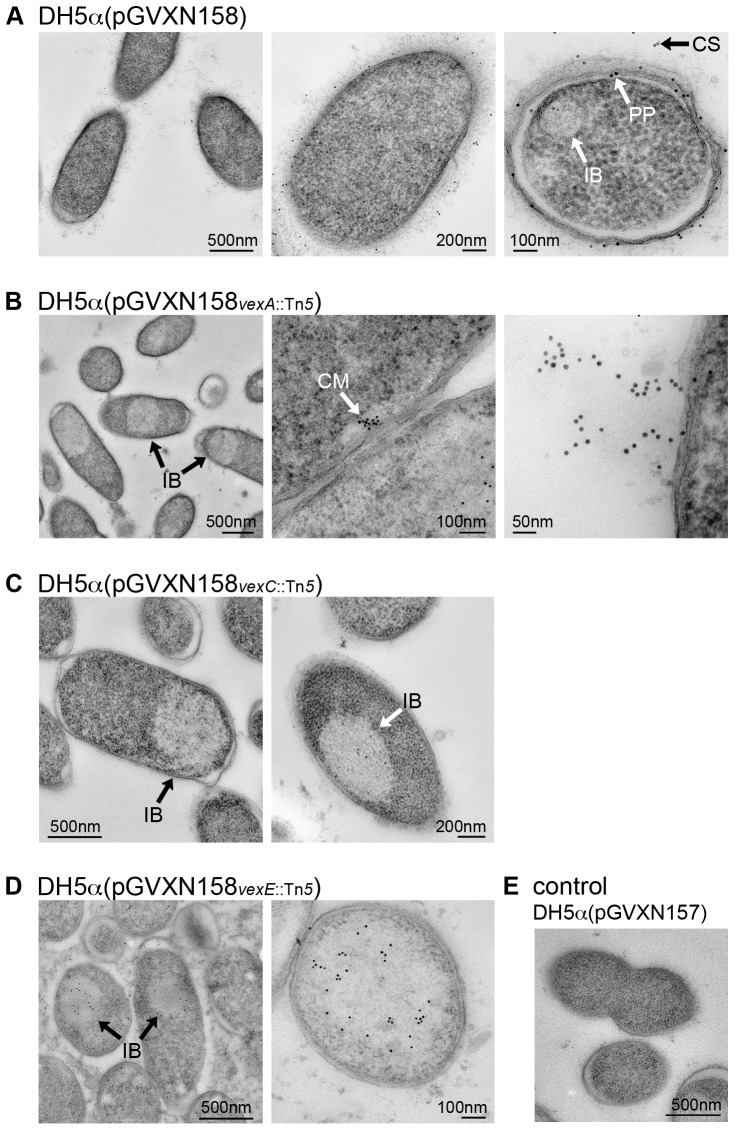
Transmission electron micrographs of ultrathin sections from *E. coli* DH5α expressing the Vi polysaccharide. The Vi has been labeled with the immunogold technique by using an anti-Vi antibody. (**A**) *E. coli* DH5α cells expressing the wild-type *viaB* operon (pGVXN158) are shown. The Vi is mainly localized on the cell surface (CS) but is also stained in the periplasmic space (PP) and inside inclusion-like bodies within the cytoplasm (IB). (**B**) *E. coli* DH5α cells containing the *viaB* operon with a transposon insertion in *vexA* (pGVXN158*_vexA_*_::Tn*5*_), the outer membrane polysaccharide export (OPX) protein, are shown. Vi was mainly stained within large inclusion-like bodies (IB) and sometimes clustered at the inside of the inner membrane (CM). Very occasionally, filamentous Vi erupting from the cell surface was labeled. (**C**) *E. coli* DH5α cells containing the *viaB* operon with a transposon insertion in *vexC* (pGVXN158*_vexC_*_::Tn*5*_), the ATP-binding protein of the ABC transporter, are shown. The Vi polysaccharide was labeled in large inclusion bodies in the cytoplasm. (**D**) *E. coli* DH5α cells containing the *viaB* operon with a transposon insertion in *vexE* (pGVXN158*_vexE_*_::Tn*5*_) are shown. The Vi polysaccharide was labeled in large inclusion bodies in the cytoplasm. (**E**) As a control *E. coli* DH5α cells containing the empty plasmid pGVXN157 are shown.

In addition to the Vi quantification and localization studies by ELISA, several selected *vex* transposon insertion mutants were analyzed by transmission electron microscopy (TEM) and immunogold labeling of the Vi polysaccharide using the monoclonal anti-Vi antibody P2B1G2/A9 [28]. The specificity of the antibody was tested by immunogold labeling of DH5α cells carrying the empty plasmid pGVXN157 (Figure 3E). These cells do not express the Vi polysaccharide and practically no staining was observed. Figure 3A shows the normal cellular distribution of the Vi antigen in DH5α(pGVXN158). The images show encapsulated bacteria where the polysaccharide is mainly localized at the cell surface, but Vi antigen is also detectable in the periplasmic space and inside inclusion-like bodies within the cytoplasm.

As expected, the Vi polysaccharide was differently distributed within the cells carrying *viaB* transposon insertion mutant plasmids. In cells harboring *vexA*::Tn*5* encoding a nonfunctional outer membrane polysaccharide export (OPX) protein, the Vi was mainly stained within large inclusion-like bodies and sometimes clustered at the inside of the inner membrane (Figure 3B). Vi accumulation at the cytoplasmic membrane might indicate the site of Vi translocation over the inner membrane or an accumulation due at a blocked pore complex. Very occasional labeling of filamentous Vi erupting externally from the cell surface was observed.

In cells expressing a nonfuctional ABC transporter ATP-binding protein VexC, DH5α(pGVXN158*_vexC_*_::Tn*5*_), the Vi was labeled in large inclusion-like bodies inside the cells (Figure 3C). Disruption of the transport motor in this mutant appears to completely block Vi translocation through the pore complex.

Cells harboring the *vexE*::Tn*5* plasmid display a similar phenotype as seen in the *vexC*::Tn*5* insertion mutant (Figure 3D). Vi was mainly stained within inclusion-like bodies inside the cells. The extracellular Vi detected by ELISA was not visualized in the extracellular spaces.

### Defect of the outer membrane polysaccharide export (OPX) protein VexA results in cell instability

**Figure 4:**
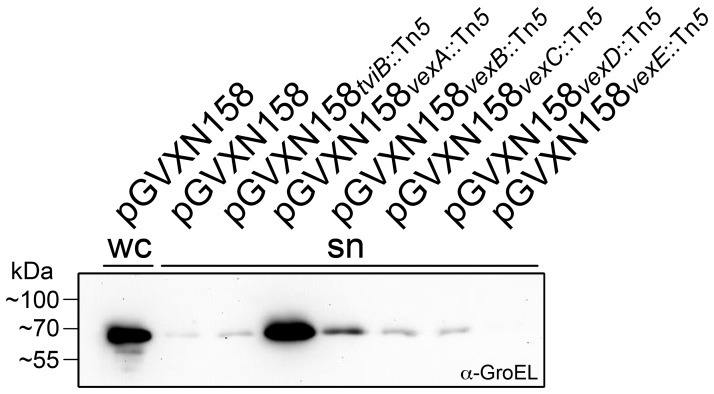
Detection of the cytosolic protein GroEL in cell culture supernatants of individual *vex* mutants. Either whole cells (wc) or supernatants (sn) of DH5α cell cultures carrying the indicated plasmids were separated by SDS-PAGE and after transfer to a nitrocellulose membrane, GroEL was detected by western blot using an anti-GroEL antibody. Supernatant samples were normalized by TCA precipitation to the optical density of the cell culture. In DH5α cells expressing the wild-type *viaB* operon (pGVXN158), GroEL is mainly detected in the cell pellet. In contrast, the supernatant of a *vexA* transposon insertion mutant (pGVXN158*_vexA_*_::Tn*5*_) contains substantial amounts of the cytosolic marker protein, indicating cell instability.

It was unexpected to detect large amounts of Vi in the cell culture supernatant and on the cell surface of *E. coli* harboring the *vexA*::Tn*5* mutant derivative. In TEM only very occasionally cell surface associated Vi could be detected (Figure 3B) whereas using ELISA and immunoblotting (Figure 2) nearly wild-type levels of surface exposed capsular sugar were detected. One hypothesis to explain these observations would be, that cells have a functional transporter in the inner membrane but lack the outer membrane pore might show potential leakiness, therefore releasing the Vi from these instable cells. To test this hypothesis we analyzed the presence of a cytosolic protein, GroEL, in the cell culture supernatant of individual *vex* mutants. Therefore, cells of an overnight culture equivalent to an A_600_ of 1 were collected and proteins were precipitated from the supernatant with trichloroacetic acid. Samples that were hereby normalized to the optical density of the overnight culture were separated by SDS-PAGE and GroEL was detected by western blot using an anti-GroEL antibody (Figure 4). As expected, GroEL was mainly detected in the cell pellet and not in the cell culture supernatant of *E. coli* DH5α(pGVXN158). There were only traces of GroEL detectable in the supernatants of DH5α(pGVXN158*_tviB_*_::Tn*5*_), DH5α(pGVXN158*_vexB_*_::Tn*5*_), DH5α(pGVXN158*_vexC_*_::Tn*5*_), DH5α(pGVXN158*_vexD_*_::Tn*5*_) and DH5α(pGVXN158*_vexE_*_::Tn*5*_). In contrast, large amounts of GroEL were detected in the supernatant of DH5α(pGVXN158*_vexA_*_::Tn*5*_) cultures. These data indicate that in this mutant derivative a propensity for cell instability does exist. Therefore, the Vi detectable in the ELISA gives an impression of surface associated polysaccharide, when in fact the Vi originated from lysed cells. This explanation is also in agreement with the inability to agglutinate DH5α(pGVXN158*_vexA_*_::Tn*5*_) cells, or infect these cells with a Vi phage.

### Size exclusion chromatography of exported Vi produced by E. coli DH5α(pGVXN158) and a vexE::Tn5 transposon insertion mutant

**Figure 5:**
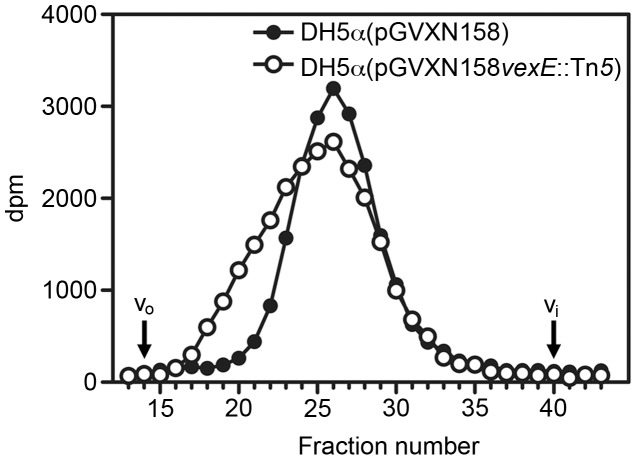
Size exclusion chromatography of exported Vi produced by wild type and *vexE* transposon insertion mutant. DH5α(pGVXN158) and DH5α(pGVXN158*_vexE_*_::Tn*5*_) cells were metabolically labeled with [^3^H]GlcNAc and the Vi polysaccharide was purified from the cell culture supernatants. Purified [^3^H]Vi polysaccharide was analyzed by size exclusion chromatography using a Sephacryl S-1000 column. The elution profiles of the [^3^H]Vi preparations are shown. The void volume (v_o_) and the inclusion volume (v_i_) of the column are indicated.

The observed inability of Vi expressed by DH5α(pGVXN158*_vexE_*_::Tn*5*_) cells to bind to nitrocellulose membranes or form a capsule once transported to the cell surface lead to the hypothesis, that this polymer might have different physical properties compared to the wild-type polysaccharide. To further characterize these different Vi polymer species we radiolabeled the Vi and separated the purified polymers by size exclusion chromatography. DH5α(pGVXN157), DH5α(pGVXN158) or DH5α(pGVXN158*_vexE_*_::Tn*5*_) cells were metabolically labeled with [^3^H]GlcNAc and Vi polysaccharide was purified from the cell culture supernatants. There was around 600–700 times more incorporation of radioisotopes into samples purified from cell cultures expressing Vi than from cells carrying the empty plasmid, indicating that the Vi polysaccharide has been labeled specifically (Table 2). Subsequently, [^3^H]Vi was analyzed by size exclusion chromatography using a Sephacryl S-1000 column. The elution profile revealed that the Vi produced by the *vexE*::Tn*5* mutant derivative is clearly distinguishable from the one produced by *E. coli* DH5α(pGVXN158) (Figure 5). The wild-type Vi appears to have a uniform size distribution and the molecular size of the Vi polysaccharide was estimated to be 2×10^3^ kDa against dextran standards. The Vi polymer secreted by the *vexE*::Tn*5* mutant derivative displayed a tailing of the peak in the earlier elution fractions indicating the presence of either longer polysaccharide chains or the presence of a polymer with a different Stokes radius.

**Table 2:** Incorporation of [^3^H] into purified samples (counts/total mg protein).

Strain	cpm mg^−1^ protein
DH5α(pGVXN157)	459.1
DH5α(pGVXN158)	325’984.4
DH5α(pGVXN158*_vexE_*_::Tn*5*_)	281’563.9

### O-acetyl content of the Vi polysaccharide

The ability of a Vi phage to infect a cell is dependent on the expression of a Vi capsule that is partially O-acetylated at the C-3 position of the *N*-acetylgalactosaminuronic acid polymer [29]. The bacterial enzyme responsible for this Vi polysaccharide modification has not yet been identified. PFAM analysis and other bioinformatics interrogations of the proteins encoded in the *viaB* locus did not identify a potential acetyltransferase gene. This suggests that any acetylase is located outside the *viaB* operon. The observation that a Vi phage is able to infect DH5α(pGVXN158) cells implies, that it is well O-acetylated. Furthermore, we speculated that the different elution profiles observed in the size exclusion chromatographic analysis of Vi produced by *E. coli* DH5α(pGVXN158) and a *vexE*::Tn*5* transposon insertion mutant derivative could be due to a different degree of O-acetylation. We therefore determined O-acetyl contents of Vi produced by *S*. Typhi BRD948, *E. coli* DH5α(pGVXN158), DH5α(pGVXN158*_vexC_*_::Tn*5*_) or DH5α(pGVXN158*_vexE_*_::Tn*5*_). Vi polysaccharide of these derivatives was purified either from the cell culture supernatant (in *S*. Typhi BRD948, DH5α(pGVXN158) and DH5α(pGVXN158*_vexE_*_::Tn*5*_)) or from cell lysates (in DH5α(pGVXN158*_vexC_*_::Tn*5*_)). The O-acetyl content of the purified Vi preparations was then measured by the alkaline hydroxylamine method of Hestrin [30]. The average values of the O-acetyl content from three separate preparations are presented in Table 3. The O-acetyl content was comparable in all different derivatives, being around 2.5 mmol per gram of dried Vi polysaccharide, which corresponds to a level of O-acetylation of around 60%. These data show that the Vi produced in *E. coli* has a similar O-acetyl content as the Vi expressed by *S*. Typhi. The undistinguishable levels of O-acetylation of Vi purified from the cell lysate of DH5α(pGVXN158*_vexC_*_::Tn*5*_) indicates that the O-acetylation reaction takes place in the cytosol and that the reaction is independent of polymer translocation. Furthermore, the inability of DH5α(pGVXN158*_vexE_*_::Tn*5*_) to form a capsule does not correlate with different levels of O-acetylation of the Vi polymer.

**Table 3:** O-acetyl content of Vi.

Strain	O-acetyl content (mmol g^−1^)
S. Typhi BRD948	2.4±0.2
DH5α(pGVXN158)	2.7±0.2
DH5α(pGVXN158*_vexC_*_::Tn*5*_)	2.5±0.3
DH5α(pGVXN158*_vexE_*_::Tn*5*_)	2.4±0.2

## Discussion

Typhoid fever remains a serious public health problem in many developing countries, with, according to conservative WHO estimates, 16 million cases occurring each year, including about 600,000 deaths. The Vi capsule plays an important role in *S*. Typhi virulence by increasing the infectivity of the pathogen and the severity of the disease. Parenteral vaccines based on purified Vi CPS have been licensed and are widely used. Yet, many aspects of the biosynthesis, export and capsule formation of this important polysaccharide remain unclear. Here, a detailed molecular characterization of the *viaB* operon, encoding the biosynthetic machinery for Vi capsule formation is reported.

### O-acetylation of the Vi

The Vi CPS is a linear, acidic homopolymer of α-1,4-linked *N*-acetylgalactosaminuronate (D-GalNAcA), 60–90% O-acetylated at the C-3. The bulky nonpolar O-acetyl groups make up most of the surface of the Vi and complete O-deacetylation eliminates the immunogenicity of this CPS [7]. The bacterial enzyme capable of introducing this modification in the Vi has not yet been described. PFAM analysis of the proteins encoded in the *viaB* operon did not identify an O-acetyltransferase domain, indicating that the gene is located outside the *viaB* cluster in the *S*. Typhi genome. This situation has been demonstrated for the phase-variable O-acetylation of the group 2 capsule of *E. coli* K1. In this bacteria the modification is catalyzed by a prophage encoded O-acetyltransferase that is not linked to the capsular cluster [31].

Clearly the *viaB* operon can be functionally expressed in *E. coli* [15], [32]. However, the Vi expressed in *E. coli* has not yet been thoroughly characterized and the presence of O-acetyl modifications was equivocal. We found that the Vi produced in *E. coli* is recognized by Vi phage types I–VII, thereby converting *E. coli* into a Vi phage susceptible bacteria. Recognition of Vi polymer by a Vi phage is dependent on the presence of O-acetyl groups at the C-3 position of the *N*-acetylgalactosaminuronate (GalNAcA) building blocks and it has been shown that phage particles have deacetylating properties [29]. A conserved protein domain carrying an acetyl esterase was found to be associated with at least one tail fiber gene for all Vi phages [33]. This implies that in contrast to the tip enzymes of K1 or K5 phages, that degrade the polysaccharide backbone, the Vi phages specifically target the acetyl modification on the sugar polymer. Besides the observed susceptibility to Vi phage infection, we could show through chemical analysis that the Vi produced in *E. coli* is substantially O-acetylated. In addition, a similar degree of O-acetylation could be measured in an intracellular Vi preparation produced by a transporter deficient mutant (DH5α(pGVXN158*_vexC_*_::Tn*5*_)), indicating that O-acetylation is taking place in the cytoplasm and that it is a translocation independent process.

### Vi productivity in E. coli DH5α(pGVXN158) is higher compared to S. Typhi BRD948

It was observed that *E. coli* cells expressing the wild-type *viaB* operon produce around 6–7 times more Vi than *S*. Typhi BRD948. This observation could be explained by the presence of the *viaB* cluster in different copy numbers in the particular expression systems. The pLAFR1 plasmid derivative that contains the *viaB* cluster is reported to be present in 5–7 copies per cell [34], whereas *S*. Typhi BRD948 holds a single *viaB* operon. Furthermore, spontaneous loss of SPI-7 in Vi-negative *S*. Typhi has been reported in stored isolates [35]. The robust plasmid borne expression of the Vi might additionally account for the difference seen in CPS productivity. The observed high efficiency of wild-type Vi polysaccharide production in *E. coli* makes this strain a good model system to characterize Vi capsule formation and an interesting Vi production system.

### tviBCDE is the minimal set of genes required for Vi polysaccharide synthesis

Our data confirm the results obtained by Virlogeux *et al.*, 1995, that TviB, TviC, TviD and TviE are required for Vi polymer assembly. Firstly, no Vi could be detected in these individual transposon insertion mutants. Secondly, intracellular Vi was identified in *E. coli* cells carrying a plasmid encoding *tviBCDE* (results not shown). The latter result indicates that the Vi polymer is assembled in the cytoplasm and that assembly is independent of the translocation machinery. Similar results have been obtained with the well-characterized group 2 capsule of *E. coli* K1. It has been shown that cells harboring a plasmid encoding the biosynthetic region-2 genes alone can synthesize intracellular polysialic acid [36]. Additionally, synthesis of full-length polymer has been shown to occur *in vitro* and in a variety of transport-deficient backgrounds *in vivo* [37], [38].

### Synthesis and export of the Vi polysaccharide seem to be coupled processes

It appears to be common to group 2-like capsules that biosynthesis of intracellular polymer is independent of the sugar export machinery. However, Vi quantification in the different *vex*::Tn*5* transposon insertion mutants that encode the CPS transporter (*vexABCD*) by ELISA demonstrated different productivity in the individual mutants. Whereas DH5α(pGVXN158*_vexA_*_::Tn*5*_) expressed almost wild-type levels of Vi, the CPS productivity in DH5α(pGVXN158*_vexB_*_::Tn*5*_), DH5α(pGVXN158*_vexC_*_::Tn*5*_) and DH5α(pGVXN158*_vexD_*_::Tn*5*_) was greatly reduced. This result indicates that synthesis and export of the Vi are coupled processes. A reduction in endogenous glycosyltransferase activity has also been shown for *E. coli* K1 cells harboring export defects [22], [37]. Moreover, the formation of a hetero-oligomeric complex consisting of both biosynthetic and export machinery has been demonstrated in *E. coli* K5 [39] and *E. coli* K1 [40]. It is possible that the predicted inner membrane associated components of the Vi transporter (VexB, VexC and VexD) interact with the cytoplasmic based biosynthetic machinery and that disruption of the transporter indirectly decreases biosynthesis of the Vi polymer by unknown mechanisms. It seems that this observed negative feedback mechanism between a non-functional exporter and the biosynthesis of Vi polymer does not exist when the outer membrane polysaccharide export protein VexA is nonfunctional. These mutant cells produce almost wild-type amounts of CPS. Furthermore, the Vi could sometimes be detected clustered against the inner membrane by transmission electron microscopy in DH5α(pGVXN158*_vexA_*_::Tn*5*_) cells, which might indicate the formation of a functional complex between inner membrane export and biosynthesis components. Nevertheless, periplasmic accumulations of Vi could not be detected in these mutant cells as it has been described for *E. coli* K1 and K5 mutations in the homologous gene *kpsD* [41], [42].

DH5α(pGVXN158*_vexC_*_::Tn*5*_) cells accumulated the Vi polysaccharide in large intracellular, inclusion-like bodies as seen by transmission electron microscopy. This phenotype has also been described in *E. coli* K1 [38], [43]. However, inactivation of the *E. coli* K1 ABC transporter protein KpsT, resulted in a slightly different localization of the K1 polymer within the cell, where it accumulated at discrete sites around the periphery of the cells, located against the cytosolic membrane.

### High Vi expression and non-functional export machinery results in cell instability

Our data clearly demonstrate that the high levels of Vi produced in DH5α(pGVXN158*_vexA_*_::Tn*5*_) cells is not cell surface exposed. These cells can neither be agglutinated using Vi specific antisera nor be infected by a Vi phage. In addition, transmission electron microscopy pictures of these mutant cells do not show a capsule but intracellular accumulations of the Vi polymer. Substantial amounts of the cytosolic protein GroEL could be detected in the cell culture supernatant, indicating that large amounts of Vi produced by this export deficient mutant destabilize the cell. The Vi detected in the cell culture supernatant and in fixed cells by ELISA and immunoblot represents therefore not translocated but rather released polysaccharide from lysed cells. In transporter deficient mutants showing reduced Vi assembly activity (DH5α(pGVXN158*_vexB_*_::Tn*5*_), DH5α(pGVXN158*_vexC_*_::Tn*5*_) and DH5α(pGVXN158*_vexD_*_::Tn*5*_)) less GroEL was detected in the cell culture supernatant compared to the *vexA*::Tn*5* transposon insertion mutant. It seems that the level of Vi biosynthesis in transporter deficient mutants correlates with cell instability. The observed destabilizing effect of intracellular CPS accumulations on cell integrity also underlines the necessity that assembly and export have to be coupled and coordinated processes. However, the observed phenotype of a *vexA* mutant might be less pronounced in a *S*. Typhi where the Vi polysaccharide is not overexpressed.

### Retention of the Vi polysaccharide on the cell surface is dependent on VexE

VexE shows no homologies to other proteins encoded in group 2-like capsular gene clusters and it is exclusively found in the *viaB* operon. PFAM analysis of VexE identified a C-terminal acyltransferase domain also found in HtrB (LpxL), an enzyme involved in Kdo_2_-Lipid A biosynthesis. Vi quantification by ELISA demonstrated, that Vi production in DH5α(pGVXN158*_vexE_*_::Tn*5*_) cells was only slightly decreased compared to *E. coli* cells expressing the wild-type *viaB* operon. The Vi polysaccharide produced by a *vexE*::Tn*5* mutant is mainly found in the cell culture supernatant as determined by ELISA. There was no Vi visualized in the extracellular spaces by TEM and immunogold labeling, but this could be lost during sample preparation. Together, these results indicate that the Vi in a *vexE*::Tn*5* mutant is efficiently transported to the cell surface, but once translocated, the polysaccharide is not retained on the cell surface but is rather secreted. Therefore these mutant cells can neither be agglutinated using Vi specific antisera nor be infected by a Vi phage. Besides the observed inability of this Vi species to form a capsule, the polysaccharide also does not bind to nitrocellulose membranes, indicating that this species has different physical characteristics compared to the wild type Vi. We can rule out that the observed properties are due to a different degree in O-acetylation because in both Vi preparations a similar O-acetyl content was determined. However, these observations might be explained by a lipid modification that is introduced by the acyltransferase-like enzyme VexE. In addition, the polysaccharide produced by a *vexE*::Tn*5* mutant displays a broader size distribution compared to the wild-type Vi on a Sephacryl S-1000 column. This lipid modification might lead to a different stoke radius, explaining the different behavior in size exclusion chromatography. It is equivocal if the observed phenotype of a *vexE* mutant plays a biological role. It might be possible that *S*. Typhi can switch between a capsule producing and a Vi secreting state during different stages of pathogenesis by regulating VexE activity. The precise function of VexE in Vi capsule formation should be addressed in future research.

### Conclusions

In summary, we conclude that the Vi capsule biosynthesis shows many characteristics that are typical for group 2-like capsules. The Vi is completely assembled in the cytoplasm by the gene products of *tviBCDE*, and is subsequently transported to the cell surface by an ABC transporter encoded by *vexABCD*, that closely resembles the transporters of the *Haemophilus influenzae* type b and *Neisseria meningitides* group B CPSs. However, several genes indicative for *E. coli* group 2 capsular gene clusters, like *kpsFU* and *kpsSC*, that are believed to be involved in CMP-Kdo synthesis and the speculative addition of a diacylglycerophosphate-Kdo to the CPS [11], are absent in the *viaB* operon. In contrast, the *viaB* operon contains a gene, *vexE*, uniquely found in this cluster and possibly involved in lipidation of the Vi polysaccharide. Despite sharing the basic mechanism of group 2-like capsule formation, biosynthesis of the Vi capsule displays features uniquely found in this cell surface structure.

## Materials and Methods

### Strains, plasmids, and culture conditions

All bacterial strains and plasmids used in this study are listed in Table 4. Construction of the plasmids is described below. *E. coli* DH5α and EC100 were grown in LB medium (10 g tryptone, 5 g yeast extract, and 5 g NaCl per liter) or on LB agar (LB medium with the addition of 15 g agar per liter) at 37°C. *S*. Typhi BRD948 [44] was grown in LB medium supplemented with 1% v/v Aro-mix (40 mg L-phenylalanine, 40 mg L-tryptophan, 10 mg 4-aminobenzoic acid, and 10 mg 2,3-dihydroxybenzoic acid in 10 ml of ddH_2_O) and 1% v/v Tyr-mix (40 mg L-tyrosine disodium salt in 10 ml ddH_2_O) at 37°C. If appropriate, the media contained tetracycline (20 µg ml^−1^), trimethoprim (100 µg ml^−1^), or kanamycin (50 µg ml^−1^). Cells containing pEXT22 derivatives were grown in the presence of 1 mM isopropyl β-D-1-thiogalactopyranoside (IPTG).

**Table 4:** Strains and plasmids used in this study.

Strain	Genotype or relevant description	Reference
*S*. Typhi BRD948	*S*. Typhi Ty2 Δ*aroC* Δ*aroD*	[44]
*E. coli* DH5α	K-12 φ80d*lacZ*Δ*M15 endA1 recA1 hsdR17*(rK−mK) *supE44 thi-1 gyrA96 relA1* Δ*(lacZYA-argF)U169 F^−^*	Clonetech
*E. coli* EC100	F^−^ *mcr*A Δ(*mrr-hsdRMS-mcrBC*) Φ80d*lac*ZΔM15 Δ*lacX*74 *rec*A1 *end*A1 *araD*139 Δ*(ara, leu)7697 galU galK* λ^−^ *rpsL nupG*	Epicentre

Plasmid	Genotype or relevant description	Reference
pLAFR1	low copy-number broad host-range cosmid cloning vector; Tet^R^	[34]
pGVXN157	pLAFR1 derivative with multiple cloning site inserted in *EcoR*I site	This study
pGVXN158	pGVXN157 derivative carrying *viaB* operon of *S*. Typhi BRD948 on an *AscI/SpeI* fragment; Tet^R^	This study
pGVXN209	pGVXN158 with *tviB*::Tn*5* insertion (pGVXN158*_tviB_*_::Tn*5*_); Tet^R^, Tmp^R^	This study
pGVXN224	pGVXN158 with *tviC*::Tn*5* insertion (pGVXN158*_tviC_*_::Tn*5*_); Tet^R^, Tmp^R^	This study
pGVXN198	pGVXN158 with *tviD*::Tn*5* insertion (pGVXN158*_tviD_*_::Tn*5*_); Tet^R^, Tmp^R^	This study
pGVXN200	pGVXN158 with *tviE*::Tn*5* insertion (pGVXN158*_tviE_*_::Tn*5*_); Tet^R^, Tmp^R^	This study
pGVXN225	pGVXN158 with *vexA*::Tn*5* insertion (pGVXN158*_vexA_*_::Tn*5*_); Tet^R^, Tmp^R^	This study
pGVXN227	pGVXN158 with *vexB*::Tn*5* insertion (pGVXN158*_vexB_*_::Tn*5*_); Tet^R^, Tmp^R^	This study
pGVXN201	pGVXN158 with *vexC*::Tn*5* insertion (pGVXN158*_vexC_*_::Tn*5*_); Tet^R^, Tmp^R^	This study
pGVXN202	pGVXN158 with *vexD*::Tn*5* insertion (pGVXN158*_vexD_*_::Tn*5*_); Tet^R^, Tmp^R^	This study
pGVXN204	pGVXN158 with *vexE*::Tn*5* insertion (pGVXN158*_vexE_*_::Tn*5*_); Tet^R^, Tmp^R^	This study

### DNA manipulations

Plasmid DNA was isolated using the NucleoSpin Plasmid or NucleoBond Xtra Maxi Plus kit (Macherey-Nagel). Total chromosomal DNA was isolated using NucleoSpin Tissue kit (Macherey-Nagel). Restriction enzymes (Fermentas), shrimp alkaline phosphatase (Fermentas), T4 DNA ligase (Fermentas), and Phusion High-Fidelity DNA polymerase (Finnzyme) were used according to the manufacturer's instructions. PCR and restriction fragments were purified for cloning using the NucleoSpin Extract II kit (Macherey-Nagel). All DNA sequencing was completed by Synergene Biotech GmbH (Switzerland) and synthetic oligonucleotides were ordered at Microsynth AG (Switzerland).

### Plasmid constructions

pGVXN158 contains the *viaB* cluster cloned into the pLAFR1 [34] derivative pGVXN157. pGVXN157 contains a synthetic oligonucleotide cassette formed from annealing of 5′- AATTGGCGCGCCCGGGACTAGTCTTGGG and 5′- AATTCCCAAGACTAGTCCCGGGCGCGCC ligated into the *EcoR*I-digested pLAFR1 therefore introducing unique *Asc*I and *Spe*I single restriction sites. The *viaB* gene cluster was amplified from genomic DNA prepared from *S*. Typhi BRD948 using the primers 5′- AAAGGCGCGCCGGAGTATCAGTGTGGGGCATAATC and 5′- AAAACTAGTGGCCATGAGTCTGAAGCCAGGAGGAATT. The *viaB* cluster containing PCR fragment was inserted into the *Asc*I/*Spe*I digested pGVXN157 therefore producing pGVXN158.

### Transposon insertion mutagenesis and screening of viaB insertion mutants

*In vitro* transposon insertion mutagenesis was done with pGVXN158 containing the *viaB* cluster using the EZ-Tn5 <DHFR-1> insertion kit (Epicentre Biotechnologies, Madison WI 53713, U.S.A.) according to manufacturer's instructions. The *in vitro* transposon insertion reaction was transformed into TransforMax EC100 electrocompetent *E. coli* (Epicentre) and insertion mutants were selected for the marker encoded by the EZ-Tn5 transposon (dihydrofolate reductase gene (*dhfr-1*)) on trimethoprim containing plates. To identify transposon insertions into single genes of the *viaB* cluster the library was screened for Vi negative colonies. Therefore a colony blot was performed. Transformants were transferred to a nitrocellulose membrane. After washing the membrane 3× with PBST (PBS, 0.1% Tween 20) for 10 min the membranes were blocked by incubating with 10% milk in PBST for 1 h on a shaker at room temperature (RT). Membranes were incubated with a monoclonal anti Vi antibody (P2B1G2/A9, [28]) diluted 1∶100 in 1% milk in PBST for 1 h. After washing the membranes 3× with PBST the membranes were incubated with a horseradish peroxidase labeled goat anti-mouse IgG (Sigma, A9824) diluted 1∶2000 in 1% milk in PBST. Membranes were developed using 3,3′,5,5′-tetramethylbenzidine (TMB) liquid substrate system for membranes (Sigma). Plasmid DNA of Vi negative colonies or colonies that appeared to have a reduced expression of the Vi capsule was isolated and the transposon insertion site was mapped by sequencing out of the EZ-Tn5 <DHFR-1> transposon using primers DHFR-1 RP-1 and DHFR-1 FP-1 provided with the EZ-Tn5 transposon insertion kit. Single gene insertion mutants of every gene of the *viaB* cluster except *tviA* were selected. In all the selected mutants the coding strand of the transposon encoded *dhfr-1* gene was identical to the coding strand of the genes in the *viaB* cluster.

### Antibody detection of Vi antigen on washed cells and in cell culture supernatants

Overnight cultures were centrifuged and the supernatant was separated from the cell pellet. The cells were washed 3× with PBS and finally resuspended in an equal volume of PBS. 100 µl of cell culture supernatant and washed cells were pipetted onto nitrocellulose, and the membranes were blocked for 1 h at room temperature in 10% milk in PBST (PBS, 0.1% Tween 20). The presence of Vi was detected by using a monoclonal anti Vi antibody (P2B1G2/A9, [28]) as the primary antiserum, followed by horseradish peroxidase labeled goat anti-mouse IgG (Sigma, A9824). Membranes were developed using 3,3′,5,5′-tetramethylbenzidine (TMB) liquid substrate system for membranes (Sigma).

### Sample preparation for measuring Vi content by ELISA

1 ml of an overnight culture was centrifuged 5 min at 20’000 g using an eppendorf microcentrifuge. The supernatant was separated from the cell pellet and used to measure Vi content. The pellet was washed 3× with PBS and divided into two eppendorf tubes. One half of the cells was fixed by resuspending the pellet in 4% paraformaldehyde in PBS and incubation for 30 min at RT. Afterwards the fixed cells were washed 3× with PBS and cell associated Vi was measured. The other half of the washed cells was lysed using B-PER protein extraction reagents (Pierce) according to manufacturer's instructions. Protein and Vi contents were measured in the cell lysate by the bicinchoninic acid assay (BCA, Pierce) and ELISA. The amount of intracellular Vi was defined using the difference in Vi measured in cell lysates and in fixed cells. The Vi content of different samples was normalized to the protein amount measured in the cell pellets.

### Vi quantification by ELISA

Vi polysaccharide content of samples was measured using a sandwich ELISA. Two monoclonal Vi specific mouse antibodies P2B1G2/A9 (IgG1, coating antibody) and P5B2D8/A9 (IgM, primary antibody) were used in the ELISA and are described elsewhere [28]. In order to saturate the binding of the Vi to the ELISA plate, the coating antibody P2B1G2/A9 was partially purified from the hybridoma supernatant using a 5 ml HiTrap Protein A HP column (GE Healthcare). Briefly, 50 ml of P2B1G2/A9 hybridoma supernatant were diluted 1∶10 with binding buffer (20 mM sodium phosphate, pH 7.5) and loaded on the Protein A column using a peristaltic pump at a flow rate of 20 ml min^−1^. The column was eluted with 100 mM citric acid pH 3.25 and 1 ml fractions were collected. The tubes destined to collect the antibody fractions contained 100 µl 1 M Tris-HCl, pH 9.0 to instantly neutralize the sample.

Flat bottom 96 well micro-titer plates (Nunc immuno MaxiSorb) were coated with 50 µl of the partially purified antibody P2B1G2/A9, diluted 1∶100 in PBS, at 4°C overnight. The coating solution was poured away and the plate was submerged and vigorously agitated in 4000 ml of wash buffer (1× PBS with 0.05% Triton ×100). This washing step was performed at least 4 times to completely wash out unbound antibody. Subsequently, the plate was dried by spinning upside down in a micro plate rotor. This washing procedure was always applied in further washing steps. Each well was completely filled with 300 µl of blocking buffer (1× PBS with 2.5% BSA (globulin free BSA, Sigma, A7030)) and incubated 2 h at RT on a plate shaker. After washing and drying the plate the sample was applied in an appropriate dilution in dilution buffer (1× PBS with 0.5% BSA). As a standard, Vi polysaccharide (Typhim Vi, Sanofi Pasteur MSD) was used in several dilutions in the range from 0–4 ng ml^−1^. 100 µl of sample and standard were applied in doublicates to the corresponding wells and incubated 1 h at RT on a plate shaker. After washing and drying the plate, 100 µl of the primary antibody (hybridoma supernatant of P5B2D8/A9) diluted 1∶100 in dilution buffer was added to the wells and incubated 1 h at RT on a plate shaker. After another washing and drying step the wells were incubated with 100 µl of secondary antibody (goat anti-mouse IgM peroxidase conjugated, Sigma A8786) diluted 1∶2000 in dilution buffer for 1 h at RT on a plate shaker. Following washing and drying the reaction was developed with 100 µl of Ultra TMB substrate (3,3′,5,5′-tetramethybenzidine liquid substrate, Pierce) for 15 min and stopped with the addition 100 µl of 2 M sulphuric acid. The bottom surface of the plate was wiped with 70% 2-propanol and the absorption at 450 nm was measured.

### Vi polysaccharide purification

Vi polysaccharide was purified by a modified procedure as described elsewhere [45]. The Vi was either purified from the cell culture supernatant (in strains *S*. Typhi BRD948, DH5α(pGVXN158) and DH5α(pGVXN158*_vexE_*_::Tn*5*_)) or from sonicated cells that were treated with proteinase K and nucleases (in strain DH5α(pGVXN158*_vexC_*_::Tn*5*_)). Vi was precipitated from the supernatant or enzyme treated cell lysate with 0.1% hexadecyltrimethylammonium bromide (CTAB, Sigma, H6269). 20 g l^−1^ celite 545 (Sigma, 20199-U) was added and the mixture was stirred for 1 h at RT in order to allow the formation of a polysaccharide-CTAB complex, which adsorbs onto the celite. The celite was poured into a reservoir of appropriate size (Extract-clean EV SPE Reservoir, Socochim S.A.) equipped with a frit (Socochim S.A.). The column was washed successively by gravity flow with 10 column volumes (CV) of 0.05% CTAB, 10 CV of 20% ethanol, 50 mM sodium phosphate buffer pH 6.0, and 15 CV of 45% ethanol to eliminate adsorbed impurities. The Vi polysaccharide was finally eluted with 1.5 CV of 50% ethanol, 0.4 M NaCl. Following elution, the polysaccharide was precipitated by addition of ethanol to a final concentration of 80% and incubation for 20 min at RT. The precipitate was washed twice with 80% ethanol prior to lyophilization. The protein and nucleic acid content of the purified Vi polysaccharide was determined by the bicinchoninic acid assay (BCA, Pierce) and UV spectroscopy respectively.

### Determination of O-acetyl content in the Vi polysaccharide

The method was used as described elsewhere [30]. Briefly, the O-acetyl groups were released by mild base treatment of the Vi polysaccharide solution. The O-acetyl groups reacted with the added hydroxylamine in alkali to form hydroxamic acid. The formed hydroxamic acid was measured by the formation of a purple-brown complex with Fe^3+^. Acetylcholine was used as a standard.

### Radiolabeling of Vi polysaccharide

Cells were cultured in LB medium supplemented with antibiotics at 37°C in the shaker incubator (180 rpm). Cultures were inoculated from an overnight culture to an A_600_ of 0.01. After 4 cell doublings, 1 µCi ml^−1^ [^3^H]GlcNAc (American Radiolabeled Chemicals, Inc., ART 0142) was added and the culture was incubated 2 h at 37°C in the shaker incubator. After incubation the cells were centrifuged and the cell culture supernatant was used to purify the labeled Vi polysaccharide as described above. After drying of the purified Vi, the polysaccharide was resuspended in 50 mM NaCl. Incorporation of radioactivity in the purified samples was measured and normalized to the protein content measured in the cell pellets.

### Size exclusion chromatography

Sephacryl S-1000 (GE Lifescience) gel filtration medium was used in a column with the proportions of 23 cm×1.7 cm (height x diameter). Chromatography was done by gravity flow using 50 mM NaCl as the mobile phase and 1 ml fractions were collected.

### Transmission Electron Microscopy (TEM)

TEM was done as described elsewhere [46].
